# Law in everyday life and death: a socio-legal study of chronic disorders of consciousness

**DOI:** 10.1111/lest.12042

**Published:** 2014-02-19

**Authors:** Simon Halliday, Celia Kitzinger, Jenny Kitzinger

**Affiliations:** University of York; Cardiff University

## Abstract

This paper addresses, from a socio-legal perspective, the question of the significance of law for the treatment, care and the end-of-life decision making for patients with chronic disorders of consciousness. We use the phrase ‘chronic disorders of consciousness’ as an umbrella term to refer to severely brain-injured patients in prolonged comas, vegetative or minimally conscious states. Based on an analysis of interviews with family members of patients with chronic disorders of consciousness, we explore the images of law that were drawn upon and invoked by these family members when negotiating the situation of their relatives, including, in some cases, the ending of their lives. By examining ‘legal consciousness’ in this way (an admittedly confusing term in the context of this study,) we offer a distinctly sociological contribution to the question of how law matters in this particular domain of social life.

## Introduction*

This paper examines the topic of chronic disorders of consciousness from a legal perspective. Our intentions underlying this deceptively simple opening statement, however, require some elaboration: what do we mean by these key phrases ‘chronic disorders of consciousness’ and ‘legal perspective'? The phrase ‘chronic disorders of consciousness’ is an umbrella term referring to severely brain-injured patients in prolonged comas, vegetative^1^ or minimally conscious states.^2^ The second term – ‘a legal perspective’ – requires a little more unpacking. Legal scholarship is now a distinctly varied business, embracing a range of methodological approaches,^3^ and the topic of chronic disorders of consciousness has been studied by legal scholars from a number of angles. Important doctrinal work, for example, has been carried out exploring relevant legal provisions (most recently the Mental Capacity Act 2005),^4^ examining, in particular, how the courts have grappled with difficult cases.^5^ Other legal scholars have explored the topic comparatively.^6^ Equally, there has been a sizeable amount of fascinating work that, from various philosophical perspectives, has assessed and critiqued doctrinal provisions and case-law developments.^7^ There has, further, been a small amount of empirical medico-legal work on this topic, surveying doctors' attitudes to the care of patients in PVS.^8^

If we ‘zoom out’, as it were, and observe this body of scholarship from a high vantage point, we can see that, in general terms, it is usually animated by a fundamental underpinning research concern: what is the significance of law for the treatment, care and the end-of-life decision making for patients with chronic disorders of consciousness? This paper, in a like fashion, is driven by this basic and important question. However, it seeks to take a novel approach to its answering. And in doing so, we will demonstrate that this basic research question is even more complex than might already have been imagined. For the paper offers an empirical analysis of the ‘legal consciousness’ of the family members of patients with chronic disorders of consciousness. ‘Legal consciousness’ – an admittedly confusing term in the context of this paper about disorders of consciousness – is a term of art within the sociology of law^9^ that is much wider in its focus than the medical conditions explored in this paper. ‘Legal consciousness’ comprises society's constructions of legality – the cultural characterisations of legality that are common currency and drawn upon when, as individuals and groups, we make sense of everyday life.^10^ To study legal consciousness is to study the background assumptions about legality that structure and inform routine thoughts and actions.^11^ An empirical focus on legal consciousness, then, like much legal research, involves an enquiry into the role of law in society – but not law as expounded by the courts or legal personnel, rather ‘law’ as constructed by society in various cultural ‘narratives’ of legality, as they are sometimes described.^12^ This paper, accordingly, focuses on the images of law that were drawn upon and invoked by family members when negotiating the situation of their relatives with chronic disorders of consciousness, including, in some cases, the ending of their lives. In this way, we present a study of law in the everyday lives^13^ of ordinary people enduring extraordinary circumstances, thus offering a distinctly sociological contribution to the question of how law matters in this particular domain of social life.

The paper proceeds in four stages. First, to provide some background and context, we offer a brief overview of the legal regulation in England and Wales (where our study mostly took place) of the treatment, care and ending of lives of patients with chronic disorders of consciousness. Secondly, we give an introduction to our data set and describe the research methods used to obtain it. Thirdly, we present our research findings. And fourthly, we then discuss them from the perspective of legal consciousness, before concluding by exploring the wider implications of our analysis for this field of medical care and considering what research agenda they suggest.

## The Law

The treatment of patients with chronic disorders of consciousness is, like all medical treatment, subject to the standards of care developed in the general law of negligence.^14^ More specifically, however, treatment decision making is governed by legislation dealing with situations in which individuals lack the capacity to make decisions for themselves. The Mental Capacity Act 2005 is the statute in force in England and Wales that sets out a legal framework for determining mental capacity and for decision making on behalf of those over 16 years old who lack the capacity to make decisions for themselves.^15^ Patients with catastrophic brain injuries leading to disorders of consciousness clearly lack such capacity and, under the Act, the senior clinician with treating responsibility (usually the consultant) therefore becomes the decision maker for such patients. The only exceptions to this would be the rare circumstances^16^ in which a patient has elected in advance to refuse consent to certain treatments by way of a legally valid and applicable Advance Decision;^17^ or the patient has granted a Health and Welfare Lasting Power of Attorney to someone so that they can give or withhold consent to treatments;^18^ or the court has appointed a Welfare Deputy with the power to give or withhold consent^19^ (though a Welfare Deputy does not have the power to refuse life-sustaining treatment).^20^ Contrary to popular belief,^21^ then, the term ‘next of kin’ has no legal status in England and Wales and does not grant any decision making power over an incapacitated patient. Family members, although not the responsible decision makers, must, however, be given the opportunity for involvement in decision making regarding their loved one's care and treatment. Clinicians have a duty to consult with the patient's family in order to inform decisions in the ‘best interests’ of the patient.^22^

Although most medical treatment decisions can be taken simply as a result of discussions between the clinicians and family and friends (and/or official advocates), the decision making process is more involved in relation to ‘serious medical treatment’,^23^ including the withholding or withdrawal of artificial nutrition and hydration (ANH) from a patient in a permanent vegetative (or minimally conscious) state. All such decisions must be brought to the Court of Protection for an exercise of its declaratory power under the Act.^24^ Under s 15, the Court of Protection may make declarations as to the ‘lawfulness or otherwise of any act done, or yet to be done’ in relation to a person who lacks capacity. The issue for the court in such cases is, strictly speaking, whether it is in the patient's best interests to *give* treatment rather than to withhold or withdraw treatment, given that the jurisdiction of the court is to grant treatment consent where the patient is incapable of doing so him/herself:

If the treatment is not in his best interests, the court will not be able to give its consent on his behalf and it will follow that it will be lawful to withhold or withdraw it.^25^

The crucial test of ‘best interests’ (which marks a contrast to some other jurisdictions where the ‘substituted judgment’ of the patient is key)^26^ is not defined in the Act. Rather, the Act gives a checklist of factors that must be considered when working out what is in a person's best interests.^27^ In addition to clinical considerations, these include taking into account the patient's prior expressed values, wishes and beliefs – for example, what the patient would have wanted for him/herself. This is why consultation with family (and friends) is essential to inform any ‘best interests’ decision. The role of families, as defined by the Act, is to provide information about the person before the loss of capacity – his or her character, beliefs, values and what his or her wishes might be about treatment and care decisions. This information contributes to the court's^28^ ‘best interest’ decision, but does not determine it. In a fairly recent (controversial)^29^ judgment,^30^ even a united family view that the relative would not want to be kept alive in a minimally conscious state was insufficient to tip the balance in favour of withdrawal of treatment when set against other factors, including the value of preserving life, which weighed particularly heavily.

## The Research Project

The research reported here is part of a larger ongoing project conducted by the York–Cardiff Chronic Disorders of Consciousness Research Centre. We draw here on a data set of more than 50 family members who have experience of a catastrophically brain-injured relative in a chronic disorder of consciousness. Ethical approval for the study as a whole was obtained from the Universities of York and Cardiff ethics committees. In-depth semi-structured, audio-recorded, interviews were carried out (between 2010 and 2013) by Celia Kitzinger and Jenny Kitzinger, and then transcribed and anonymised before being shared with other members of the research team.^31^ Participants were recruited through advertising via brain-injury support groups and websites, and through social networks (the two interviewers have a severely brain-injured sister), through contacts made after giving formal presentations about our research and via care homes and snowball sampling. The study subsequently received NHS approval (from Berkshire Research Ethics Committee, REC reference number: 12/SC/0495) and we were also able to recruit via consultants, although all interviews took place off NHS premises (generally in people's homes). Interviews were mostly one-to-one, but occasionally in pairs (e.g. a husband and wife asked to be interviewed together, as did a mother and daughter). Interviewees were mostly parents, siblings, spouses/partners and adult children of the patient. Most patients were currently either PVS or MCS (some had died by the time of interview; others had emerged from chronic disorders of consciousness with severe neurological deficits).

The recruitment methods used clearly do not result in a sample representative of all families with severely brain-injured members. Although the pool of interviewees^32^ shows considerable variation in terms of age, gender, ethnicity, and cultural and economic capital,^33^ we can make no claim as to the representativeness of our sample in relation to the sampling frame (i.e. all those with relatives with a chronic disorder of consciousness). Equally, as a purely qualitative study, we can make no claim as to the distribution of various legal consciousness narratives amongst those with relatives with chronic disorders of consciousness. Nor do we claim that legality was the dominant theme of our interviews. Interviews were often quite long and involved discussion of many issues. Indeed, in some interviews issues of legality were not canvassed positively at all. But although the study of legal *unconsciousness*, as it were, could be as important and revealing as that of consciousness,^34^ our focus here is on the interviewees who positively communicated perceptions of legality. This permits us to discover the significance of legal consciousness for the thoughts and actions of these interviewees in relation to their relatives. This kind of grounded analysis, in turn, allows us inductively to hypothesise about the potential significance of legal consciousness more widely in this domain – findings that can be tested and refined in further work. In other words, our data set allows us to build theory about the potential significance of law for chronic disorders of consciousness. Given how little research exists with families of these patients, and that this analysis is the first to explore legal consciousness in this field, we believe that the theory-building in this paper is a very important step.

The conduct and analysis of the interviews that gives rise to our findings followed the broad methodological trend within legal consciousness work.^35^ Where possible, the direct questioning of interviewees about law was avoided. Instead, the focus was upon the characterisations of legality that emerge naturalistically in the ways in which participants discuss their lives and actions generally, or certain topics specifically. Thus, our interviewees, in discussing the situation of their relatives with chronic disorders of consciousness and the approaches taken to their care, treatment and death, revealed their assumptions about legality – assumptions that informed their views and actions. It is these assumptions – aspects of broader cultural narratives of legality – that are the focus of our analysis. It is to this analysis that we now turn.

## Research Findings

The clearest and perhaps most obvious finding from our data – one, no doubt, that can be confidently projected inductively on to *all* those who find themselves in these circumstances – is that the experience of a relative suffering a severe brain injury is a shocking one that propels family members into a state of great uncertainty. Neurology is a complex field of medicine, beyond the ken of most laypeople. As in many fields of medicine (perhaps more so than many), family members found themselves initially entirely dependent on the expertise of medical staff. For example, one interviewee, Kim (all names of people and places are pseudonyms), noted that:

… when we started this, I was such an innocent and if somebody had said to me ‘Right, do we operate or don't we? Do we put him into intensive care or don't we?’ … I wouldn't have actually known. I was very much in the hands of the professionals …

Another interviewee, Gill, expressed a similar sentiment:

you rely on these people who are at the top of their fields to make these decisions and so you trust them.

Gill's statement about the inevitability of initial trust reflects a common assertion within the broader sociological literature on trust. The medical system with which family members find themselves having to engage is, in Giddens' terms, an ‘expert system’^36^ – perhaps, indeed, *the* expert system, *par excellence*. It is opaque and confusing for most laypeople. Trust in the medical knowledge and expertise of trained staff is the antidote to the initial sense of uncertainty felt by families.^37^ As Sztompka has noted in relation to the role of trust in contemporary society:

More often than ever before we have to act in the dark, as if facing a huge black box, on the proper functioning of which our needs and interests increasingly depend. Trust becomes an indispensable strategy to deal with the opaqueness of our social environment. Without trust we would be paralysed and unable to act.^38^

However, this initial trust can be short-lived. Indeed, such was the case for a number of our interviewees. There are, we suggest, several features associated with chronic disorders of consciousness that reduce the likelihood of initial trust in medical expertise enduring undiminished. For a start, there are the limits of medical expertise in relation to severe brain injury, and associated levels of uncertainty about outcome in the early time period. From the outset, clinicians may thus explicitly inform families of the limits of medical knowledge about, and ability to intervene in, severe brain injury, making comments such as ‘time will tell’ and ‘we have to wait and see’. Secondly, medicine may be implicated as a *cause* of the disorder of consciousness (e.g. resulting from surgery going wrong or ‘half-successful’ efforts at resuscitation). Over time, there may also be concerns from family members (and tensions between families and funders/care providers) about what care and rehabilitation can be provided, or what options are available to relieve suffering and distress, or indeed, to allow death.^39^

Another distinct feature of disorders of consciousness that may have a profound implication for trust in medical expertise is the fact that patients have little or no ability to communicate about anything they may be experiencing. This means that family members become involved in interpreting non-verbal signs as part of the wider process of deciding how best to care for the patient and what is in the patient's ultimate interests. And, of course, family members draw from their long-term and intimate knowledge of their relative, including their sense of what their relative would have wanted – knowledge that medical staff do not share. In a significant reversal of the trust–expertise dynamic, many of the family members we interviewed felt that *they* were the experts about the patients' experiences of treatment and care and that the medical staff should trust *them*, not the other way round.^40^ Gill, for example, who, above, noted her initial trust in the medical staff caring for her partner, Oscar, very poignantly described her ultimate lack of trust in the staff's understanding of him:

It was really bugging me that they were just sedating him and not actually going to the root of the problem. And because they were saying, ‘we are neurology nurses, we know seizures when we see them’. And I said … ‘you may know neurology patients and you may know seizures, but I know Oscar. And he's not having seizures.’

Likewise, Sarah raised this issue in relation to her family member's treatment – a young man supposed to be ‘vegetative’ (and thus without any awareness of himself or his environment):

They would do all of these things but not look at Ricky and see that he was in pain because, ‘Oh, he can't feel pain.’ And when they tell you that and you know that you've seen it, you think, ‘My God, how can you?’ Again, you're back to what your nightmares are made of. How can you trust? You've got to entrust them with them and you've got to walk away and leave them.

Sarah's statements point to the intimate relationship between trust and risk, another key theme in the sociology of trust. Trust is usually depicted as a strategy for managing risk.^41^ However, the flip-side of this dynamic, as we can see here, is that where trust diminishes one is left with the sense of risk. Where family members no longer trusted medical staff to fully understand their relative, our interviewees perceived their ongoing care as a source of risk – risk that their best interests would not be promoted and that avoidable suffering would be endured. Perhaps inevitably, this often became a source of tension between family members and clinicians. It is in the negotiations of these tensions that we gain our first insight into the significance of legal consciousness. The first of three key images of legality that form part of different legal consciousness narratives becomes apparent in this context.

## Law as Sword

A key theme to emerge from our data set is that family members felt embattled in relation to the care of their relatives. Many felt they had to fight to achieve what, in their view, was best for their loved ones. Hugo, for example, described a process of struggle in relation to his wife:

It's been a long, long battle trying to find the right thing … I won't say all the things get resolved but … generally … it's made a bit better … It is a long … daily process of just making sure that everything is okay.

Likewise, Elspeth described the difficulties her family experienced in trying to secure her brother's transfer to another hospital where they felt his needs would be better catered for:

That's how we got into St Peter's Hospital basically. It's from resources and arguing and bullying, basically, just by not letting up. And then also by having friends who are doctors who knew someone who knew someone.

It is important to note that most interviewees experienced the medical system as a powerful one, and many perceived themselves as having a comparative lack of power in relation to it. Some interviewees, indeed, felt belittled by their experiences. Tracy, for example, expressed this sentiment forcefully:

I sit here, I'm like a witch sitting here and now, what can I do next? How do I handle these people? They are dreadful. They are so precious. They are so territorial. They are so – because, you see, they think we're dog shit …

Equally, Elspeth, in discussing her anger about her family's struggle for the care of her brother, expressed her sense of not having been respected:

Int: Do you think any of that anger was legitimate as opposed to just expressing pain? Are there people you ought to have been angry with?

Elspeth: Angry at doctors who didn't listen to us and treated us like idiots … Yeah, angry at doctors for not listening.

However, as we saw above in relation to Elspeth, some interviewees, despite their frustrations, felt they had the resources, skills and determination to challenge decisions that were being made by medical staff. But others turned to law in order to redress the power imbalance. Tracy, for example, sought legal assistance when she felt that medical staff were not being sufficiently open with her about what had caused the minimal consciousness of her partner, Trevor:

I phoned [the solicitors] and they said to me ‘it sounds like either there was a mistake made … or maybe they did everything they could and we're just really, really unfortunate. But when you get him out of Estridge Hospital get his records, send them down to me and I'll look at them and I'll tell you what the story is.’

Likewise, Gill expressed her faith in law to mitigate a power imbalance. In her case, she was worried that life-sustaining treatment might be denied to her badly injured partner and turned to the law to try to prevent this:

… you can take [disagreements about care decisions] to your lawyer, you can get your lawyer involved. I tell you what, that put them on edge … I said … ‘I'm just going to go and run this past my lawyer.’ (laughs) Do you know, like I was feeling threatened, so I was using her as my power.

For people like Gill and Tracy, legality acts like a sword against the power of the medical system. Locked in a struggle over the welfare of their relatives, law can be turned to in order to alter power relations and influence decision making about treatment and care. Gill's decision to phone a human rights organisation for legal help suggests a perception of law as a weapon of justice and as representing some kind of higher normative order. Likewise, when Tracy described her turn to law as way of trying to prevent poor treatment happening to other patients in the future, legality is being imagined as a means of combatting unfairness and injustice in the medical system.

Yet Tracy's interview displays some ambivalence about the nature of legality as a weapon, suggesting it can be something of a double-edged sword. She additionally framed her turn to law, not as a way of promoting general fairness or respect for human rights but, rather, merely as a way of securing damages under the law of negligence so that she could pay for better care for her partner, Trevor. Equally, she contradicted her earlier concern for the situation of future patients:

my brother in law Karl, he's a lovely fellow but he tends to get emotional, and he was saying ‘this is a dreadful situation, there's … people out there who need this [information]’. And I was going ‘yeah, Karl that's for another day, okay? Let's concentrate on Trevor. I don't give a fuck about those other people to be quite honest right now.’

Here, the image of legality, albeit temporarily and perhaps to a limited extent, is disconnected from justice and becomes merely a powerful means to an end. Law is less an expression of a collective justice, and more a tactical weapon to be wielded instrumentally in a personal struggle.

## Law as Shield

Both Gill and Tracy were speaking to the interviewers within a year or two of their partner's initial injury, and both believed that the partner would recover from their chronic disorder of consciousness and were fighting to keep them alive and to secure the conditions that would help them improve. Not all of our interviewees however, shared this belief. A number of them believed that their relatives' disorder of consciousness was irreversible and that it was in their best interests to die (even if they had earlier taken a view more like Gill's or Tracy's). For some of these interviewees, the ‘wait and see’ period was long over and they no longer hoped for recovery as the means of ‘release’ but saw death as the only way forward.^42^ Here, at least for some, legality could act as a shield. Lillian and Kim were two such interviewees. Both had applied to the Court of Protection in England and Wales for a declaration that it would be lawful to have artificial nutrition and hydration withdrawn from their relative, resulting in their deaths. For both, the fact that this decision was being made by a court was highly significant and helped to protect them from feelings of responsibility. Lillian's relative had suffered severe brain injury after an operation. Before that operation, he had written a letter expressing his desire to refuse life-sustaining treatment if he was rendered incapable of making a decision for himself. However, this letter did not meet all the criteria necessary to be compliant with the requirements of an Advanced Decision under the Mental Capacity Act 2005.^43^ Nonetheless, the Court of Protection, in part informed by this letter, made a declaration that it was lawful to allow artificial nutrition and hydration to be withdrawn. The court's jurisdiction to decide this was highly significant to Lillian:

I think the fact that he'd written what he wrote helped you cope with it in your head. Because otherwise it would feel like it was more your decision … If the Court of Protection wasn't there to say, ‘Well *we* are making the ultimate decision and this is what *we* decide’, I would always feel that it was me who'd actually chosen to do it … almost feeling that you'd sentenced them to death … So the Court of Protection has shielded me from that experience.

Kim expressed similar sentiments in relation to the Court of Protection's decision about her son:

Kim: If you make a decision to end a life and then … somebody changes their mind … if you don't have a court ruling for it, then the ensuing recriminations could destroy a family … Whereas if … you're thinking ‘we decided this was a good idea, it then went through due process of law and it was looked at by someone who had no emotional involvement at all …’ then it takes the … guilt … out of it.

Int: Did it feel like it was the court's decision, not yours ultimately?

Kim: Exactly. Yes. And that's what I was told. ‘You're not deciding to end your son's life. You are posing a question that the judge will then answer for you’ …

Here, the image of legality, encapsulated in the Court of Protection's jurisdiction, is one of impartiality – ‘no emotional involvement’, as Kim put it. Both Kim and Lillian were relieved to be able to pass this life-and-death decision up to a higher decision making forum.

In relation to both Kim and Lillian, the image of law as a kind of shield was invoked in relation to their own feared sense of individual responsibility. However, Kim's reference to ‘due process of law’ connects with a wider sense of law as a shield that was also evident in our data. In other interviews, the benefits of due legal process were referred to in relation to society protecting its members from reckless decision making in relation to those with chronic disorders of consciousness. Here, legality is an impartial and appropriate form of authoritative collective regulation that merits compliance and respect. A number of interviewees displayed considerable deference to formal state legality in this regard. Jim is one such example. Jim's interview is of particular interest because his sister, who had been diagnosed as being in a permanent vegetative state, had been killed by his mother after delays in the legal process of being granted permission to withdraw artificial nutrition and hydration. A doctor appointed by the Official Solicitor to give a second opinion on Jim's sister's condition had raised the possibility that she might be in a chronic minimally conscious state, rather than in a permanent vegetative state:

When that last … report was produced by Doctor Smithers that clearly was going to delay proceedings further … it was clearly not reaching a conclusive situation, because Doctor Smithers' report was just going to kick it all into touch again. So then … my mother had reached some decision in her mind and she borrowed someone's insulin, and there was a [hospital] car that was adapted for taking a wheelchair. You can borrow it and sign it out … And you have to have somebody else with you, a third person to look after them in the back of the car … But she … didn't fill in the form or ask for anyone else to come along – she just went and asked for the keys and they gave them to her (laughs). She was so well known there, and it's a failure of procedure, but anyway. She took the car, took her home, and they were both found dead later on that day. (In accordance with our anonymising strategy outlined in n 31, certain details have been changed)

Yet, despite Jim's support for his mother's decision, he was nonetheless very deferential to legality:

These are momentous decisions that need to be done by a disinterested, authoritative and experienced party … If you have a legal system, you can't have people taking the law into their own hands … I still support the fact you've got to have a system, you've got to stick to it.

The deferential approach that Jim displayed towards legality can be significant for other family members contributing to decisions about whether and how to allow their relatives to die. John is one such example. John and other members of his family had asked the NHS Trust to apply to the Court of Protection for a declaration that it would be lawful to withdraw artificial nutrition and hydration from his wife who was in a permanent vegetative state. He was critical of the fact that his wife's consultant had not made the possibility of this legal process known to him. He had learned about it, instead, from a television programme. At the time of interview, the court hearing had been provisionally scheduled for a couple of months' time. Once involved in the application process, however, his sense of it was that it was a procedural formality with a foregone conclusion:

Int: What do you think will be the outcome of the case? Do you have any sense of whether it would be approved or not?

John: Well I think it will be, because the Official Solicitor's involved, so I think the legal bods will get together and they'll say it's a no contest this and they'll just nod it through … One reason why it might be a little more protracted than normal is they want to hit it on the bounce in that they don't want it to go to court and then come back and more work to be have to be done.

In John's view, the decision to allow a dignified end to his wife's life had taken too long. And he acknowledged the role of law in lengthening that process:

Of course when you get the legal people involved, everything then slows down for whatever reason … We've got all the reports from the senior clinicians and then the legal people have got to put that into legal speak to present it to the court … It might take quite a long time before it gets heard by the judge … it'll be a year since we started the process … and it'll be … four years since she's been in this state. So it's taken five years.

Yet, despite the fact that the Court of Protection proceedings that caused further delay to his wife's death was a legal requirement,^44^ and despite this process being a matter of ‘nodding it through’, he was still deferential to legality and was content to see due process complied with:

Fundamentally, I think it should go to court … At the moment, the clinicians know what the law says so they abide by it which means that we have to go through this long protracted process … There need to be safeguards and safety checks.

Not all our interviewees however, displayed this kind of deference to law in light of its role in protecting society from reckless decision making. For others, law was not a shield in this sense but, rather, was an illegitimate barrier to ending what they perceived to be their relatives' indignity or suffering. Like Jim's mother, above, some of our interviewees were willing to subvert the legal process. It is to these data that we now turn.

## Law as Barrier

Within our data set, in addition to deference towards legality, there was also, conversely, considerable scepticism towards the legal regulation of the ending of patients' lives. Death can only be allowed through the withdrawal/withholding of treatment that may, in some circumstances, include the withdrawal/withholding of artificial nutrition or hydration. However, where a patient has a diagnosis of being in a permanent *minimally conscious* state (MCS) rather than permanent *vegetative* state (PVS), the courts have not so far been willing to grant permission for the withdrawal of artificial nutrition and hydration.^45^ In this context, some of our interviewees expressed a willingness to kill their relative themselves – either because they thought a court would not allow their relative to die (because of an MCS diagnosis) and/or because they viewed the legally permissible route of ANH withdrawal to be intolerable and lacking in compassion. But for such people, the act of killing would not involve ‘taking the law into their own hands’ as Jim, above, put it. Rather, it would be a legitimate subversion of legality because of its illegitimacy in this context. This orientation towards legality involves an external and critical stance. Rather than showing deference to law because of its authoritative status in regulating collective matters, some interviewees observed law from the outside, as it were, and critiqued it for its normative failures. This was expressed in terms of law failing to meet acceptable moral standards. Elspeth, for example, discussed the situation of her brother, Ian, who had been diagnosed as MCS. Although Ian eventually died of natural causes, prior to this Elspeth had been planning to kill him:

Elspeth: … he was in so much pain, breathing really difficult and I said ‘Ian, I just wish there was something I could do.’ And he again leant out and looked at me. And to me that meant ‘there is something you can fucking do’ … And it just – it suddenly became really clear that that's what we had to do is to help him do that. And when we went to see the lawyer and it basically looked impossible [to win a case for ANH withdrawal], having rationalised it and realised that this was the best thing for him, I was personally wanting to take his life illegally …

Int: But you'd have faced a prison sentence.

Elspeth: Yeah, but that would be not as bad as his sentence … If we know that something is the right decision to do then it wouldn't be something I'd question massively afterwards …

Indeed, for Elspeth, not only was the law morally wrong in relation to the ending of her brother's life; it was an inappropriate intrusion into this domain:

There needs to be an option somehow. I mean, obviously you can't just be, ‘Hey he's MCS, let's off him.’ But I don't think it should be a legal thing. I think it should be down to the doctors with the carers and it should be a multidisciplinary thing. I don't think it *is* a legal matter.

A similar scepticism towards legality was expressed by Sarah. Like Jim, her family member had been killed by another relative. Unlike Jim, however, Sarah showed little deference towards the law in this regard. Indeed, she had been willing to do the same:

I always said I don't think it'd be wrong, but I am not going to let that system get hold of me. I couldn't deal with prison. I mean I'd end up probably killing someone else as well (laughs) and be truly a murderer … In some cases, they say it's wrong what Mary did. Well, morally it isn't wrong … And I don't care what the law says.

Sarah's reference to being ‘truly a murderer’ suggests that, from her perspective, formal legality has only a contingent relationship to justice. The legal category of murder is only ‘true’ when it corresponds to a higher normative order. Where it doesn't, then the law, lacking legitimacy in this respect, can be ignored and subverted.

## Discussion

What should we make of these different images of legality identified from our interviews? Two potential preliminary objections to our analysis should be anticipated and countered before the discussion ensues. First, it is tempting, perhaps, to view our analysis not as one of law itself but, rather, as one of *attitudes to* law. One quick response to such a concern would be to note that attitudes to law – the focus of much socio-legal work over the years^46^ – are as worthy of study within the legal academy as legal doctrine itself. However, the deeper point of the legal consciousness literature is that if we are concerned with the rule of law in society, we cannot avoid examining how legality is constructed *by* society. For the societal constructions of legality away from the formal sites of law – law in everyday life, in other words – *constitutes* the rule of law in an important sense. As Ewick and Silbey have noted in an interview about their legal consciousness research:

The law is what people do about the law. We said that people's engagement with the law in their lives was an ongoing construction of relations. Law was just a flavor to any social relation. So in order to understand the rule of law we had to find its place in ordinary social relations. So the question was not only ‘what do people do?’ It was also ‘what is the rule of law?’^47^

The study of legal consciousness, therefore, is as much the study of law as is the study of legal doctrine. In this way, our paper makes a novel contribution to our wider understanding of how law matters in the care of those with chronic disorders of consciousness.

Secondly, it might be objected that an analysis of individuals' attitudes and actions does not sustain the claim that we are studying legal consciousness narratives as such – society's constructions of legality. Our response here is that, although individuals have attitudes, attitudes are not individual. Orientations towards legality are social rather than individual. The contribution of the legal consciousness literature has been to highlight the ways in which legality is *socially* constructed in society. And such constructions are not infinite. The work of both Ewick and Silbey^48^ and Halliday and Morgan^49^ (discussed below) contends that the constructions of legality within society are limited and capable of structured analysis.

It is important to stress again at this point that our aim in this paper is to build theory about the significance of legal consciousness for the thoughts and actions of families going through this situation. We use our data to make links between the wider body of literature on legal consciousness and the experience of having a relative with a chronic disorder of consciousness. By interrogating our interviews for images of legality, we can open up a dialogue between this data and broader legal consciousness theory. And in turn, legal consciousness theory, applied to and tested in our data, can deepen our insights into the experience of responding to the severe brain injury of a partner, spouse, offspring, parent or sibling. What we will show in the following section is that the images of law as sword, shield and barrier can be interpreted meaningfully through the lens of existing legal consciousness theory. And in light of these connections, we can hypothesise about the wider role of legal consciousness in this domain of social life.

## Legal Consciousness Theory

Ewick and Silbey proposed an influential typology of legal consciousness ‘narratives’, as they put it – separate characterisations of law in society which, they suggested, are drawn upon and reproduced in a routine fashion in commonplace lives.^50^ They name these three narratives according to the characteristic orientation towards law implicit in the narrative: (1) ‘before the law’; (2) ‘with the law’; and (3) ‘against the law’. Each narrative has a double face, as it were, representing both a characteristic individual response to law *and* a cultural schema that make sense of law at a structural level. Standing ‘before the law’ captures an image of law as ensuring collective fairness, equality and justice. Playing ‘with the law’, by way of contrast, is a story where law is a morally neutral game that can be played to individuals' advantage if they are clever enough and have the right resources. Being (up) ‘against the law’ tells yet another story of law where it is the expression of brute power, exercised unpredictably and resisted by individuals where cracks in that power appear (though no attempt is made to alter the power structures themselves).

Halliday and Morgan^51^ have recently mapped Ewick and Silbey's typology on to a broader analytical framework derived from Mary Douglas' grid-group cultural theory.^52^ Ewick and Silbey's three narratives correspond largely to three of the four ‘cultural biases’ suggested by Douglas. However, this mapping exercise revealed that a fourth narrative, corresponding to the fourth cultural bias in Douglas' scheme, is missing from Ewick and Silbey's account of legal consciousness. Halliday and Morgan applied the fourth cultural bias to the topic of legal consciousness in a study of radical environmental activism. Within this narrative of legality (which they term ‘collective dissent’), state law is similarly regarded as illegitimate and oppressive, but is resisted and subverted in a collective effort to alter the power structures that legality imposes.

Existing scholarship on legal consciousness, then, offers us four core cultural characterisations of legality:^53^ (1) before the law; (2) with the law; (3) against the law; (4) collective dissent. Our suggestion is that the images of legality revealed in our data are rooted in these four cultural narratives.

### Before the law

The image of law as both a sword and a shield connects largely with Ewick and Silbey's ‘before the law’ narrative of legality. The idea of law being a powerful weapon of justice to counteract and call to account the failings of the medical system is part of the story of law as ‘a general, objective and impartial power’,^54^ as Ewick and Silbey put it. Gill's sense that formal law was protective of human rights, and Tracy's instinct to invoke law to protect the interests of others, correspond to the story of law as a reified system of justice. As Ewick and Silbey noted:

Individuals' decisions to mobilize the law thus often involved the crucial interpretive move of framing a situation in terms of some public, or at least general, set of interests.^55^

This same interpretive move is seen in the image of law as a shield, protecting society from reckless decision making about the ending of lives. Despite John's criticisms of the slowness of the legal process in relation to his wife's case, and despite Jim's sympathy for his mother's killing of his sister, both portrayed Court of Protection proceedings as an essential system for collective protection. The Court of Protection offered ‘safeguards’ and a demonstration that the issue would be ‘dealt with in a balanced way’ (John), and represented ‘disinterestedness, authority and experience’ (Jim). In their view, the court was the proper place for decisions about the ending of lives. In this narrative of legality, law deserves respect and compliance from deferential subjects.

### Against the law

Yet, in contrast to the deferential orientation of subjects invoking the ‘before the law’ narrative, the image of law as an illegitimate barrier connects with Ewick and Silbey's ‘against the law’ portrayal of legality, where the power of law is resisted. In contrast to Tracy's and Gill's faith in the normative qualities of legality, whereby it can ‘protect [relatives'] needs’ (Gill) or prevent doctors from ‘doing the same again [to others]’ (Tracy), the interviews with Elspeth and Sarah reveal considerable scepticism towards it in this domain. For them, given the court's reluctance in the case of *W v M and Others*^56^ to permit withdrawal of ANH in relation to a minimally conscious patient, the requirement of legal proceedings was a problematic obstacle to, and unwelcome intrusion into, the resolution of their families' suffering. As such, the demands of due legal process did not merit compliance or respect but, rather, invited avoidance and resistance – ‘the legal thing, that's what's made me most angry’ (Elspeth); ‘I've got absolute contempt for [this legal system]’ (Sarah). And despite the power of law to criminalise and punish – indeed, perhaps because of it – we saw such an act of resistance in the action of Jim's mother in her dual ‘mercy killing'/suicide. As Ewick and Silbey have noted:

Resistant acts are almost always opportunistic, dependent upon a crack or opening in the face of power … [T]hese acts are often practiced to escape, rather than change, a structure of power …^57^

Jim's mother identified such a crack in the face of power – allowing her to take her daughter out of the hospital unaccompanied and thus to administer a lethal dose of insulin. And in killing herself with her daughter, Jim's mother performed the ultimate act of escaping law's power – albeit at the cost of her life.

### With the law

Although we suggested above that the image of law as a sword is part of the ‘before the law’ narrative, it can also connect with Ewick and Silbey's ‘with the law’ narrative of legality, as we saw in relation to Tracy's interview. Here, formal law is disconnected from justice and is portrayed simply as a resource that may be harnessed tactically for individual gain. Tracy's ambivalence about the character and promise of law hints at this more instrumental and profane story of legality. In the midst of a harrowing and lengthy struggle with a powerful medical system, it is not hard to imagine such a narrative of legality being invoked.

### Collective dissent

The interview with Sarah reveals that the act of killing a loved one may be more than an act of escape from, or avoidance of, law's power, as was the case with Jim's mother. Sarah contrasted herself with her family member who had killed their loved one:

I know what a crusader Mary can be. She always has been. She might want to make a crusade of this issue with Ricky, but I don't. I mean I'm not saying I don't, but not with my life in prison, thank you … I could not have dealt with them getting hold of me. So I would've done it sneakily.

Whereas Sarah was inclined, like Jim's mother, to *escape* the power of law, others may act in order to *change* the power of law in this domain for the benefit of all those who may suffer similarly. In this contrast between Sarah and Mary, we can see a glimpse of Halliday and Morgan's fourth narrative of legal consciousness – what they call ‘collective dissent’.^58^ Here, the authority of state law is rejected and critiqued in the name of some kind of group interest. The act of killing a loved one, then, may be prefigurative or be part and parcel of a wider collective voice of dissent against the power of law in this domain.

## Conclusion

This paper has offered a socio-legal analysis of chronic disorders of consciousness. Like other aspects of society that have been studied through a legal consciousness lens (such as social welfare,^59^ workplace relations,^60^ sexuality,^61^ prostitution,^62^ radical activism^63^ and, indeed, everyday life generally^64^ ), we have demonstrated the pertinence of legal consciousness theory for legal study in this field. If, as legal scholars, we wish to understand the significance of law for the treatment of those with chronic disorders of consciousness, then we must study legal consciousness as much as we study legal doctrine. But what are the wider implications of our analysis for this field of medical care, and what research agenda do they suggest?

It is trite to stress that law surrounds and permeates this field. From above, as it were, the law seeks to regulate who gets to make treatment decisions when patients lack capacity. Further, when it is thought that the withdrawal of treatment may be in the patient's best interests, the law ultimately reserves that judgement for itself (through the medium of the Court of Protection). From below, the law is invoked by individual family members to protect their relatives' interests and to call to account a powerful medical system. One of the main insights of our analysis is that the power of law to achieve these objectives depends on legal consciousness. And it is the legal consciousness of two key groups who stand either side of the injured individual – family members and health professionals – that is central here.

In relation to family members, our data revealed that legal consciousness can undermine the capacity of law to control end-of-life decisions. They could equally undermine its capacity to support, empower or vindicate family members who feel aggrieved about the medical treatment provided to their relatives. The key questions here are about the conditions under which different narratives become salient for family members at key moments. Individuals will not sustain a single narrative in relation to all aspects of their lives. We should expect people to display a certain amount of incoherence in their legal consciousness.^65^ Equally, individuals will not sustain a single narrative over time. We should expect a certain amount of inconsistency in this respect. Although the reasons for individual orientations towards law may be complex and, in some situations, beyond full explanation, the impact of key events or interventions will, in many situations, be capable of analysis. What role, for example, do legal advisors or advice networks have here? Equally, what potential do support groups have to foster a collective sense of agency to try to alter the power structures of law in this domain, and how might such a sense of agency be lost? Some social movements, such as the civil rights movement in the USA, have been successful in challenging the power structures of law.^66^ Equally, some movements in the health domain have been successful in challenging the domain of medical expertise.^67^ However, at the same time, as Halliday and Morgan have argued,^68^ there is a significant empirical dynamic between the sense of collective agency within such activism and the individual sense of fatalism characteristic of the ‘against the law’ narrative. Empirically, collective initiatives and organisations are vulnerable to failure. Individuals who were once energised as part of a group effort to challenge the power of law can be vulnerable to shifts towards a more isolated sense of fatalism where symbolic acts of resistance rather than collective struggles are more common. We should expect such in this context too. But what are the particular features of this context that militate for or against collective agency? Longitudinal research that could reveal such dynamics would be very useful here.

Of course, our analysis of family members' accounts serves to highlight an important gap in our understanding and the need to include medical staff in future legal consciousness research. For when family members invoke the law against medical staff, its power to influence those staff depends on *their* legal consciousness. In what ways, for example, is law resisted, deferred to or played with in medical decision making around chronic disorders of consciousness? If staff draw upon a ‘with the law’ consciousness, they will probably respond tactically to the prospect of legal accountability, much like a move in a game. If they draw upon an ‘against the law’ consciousness, taking a fatalistic stance to law's power, they may resist its power where opportunities arise. Equally, they may engage in collective efforts to subvert or alter the power of law.^69^ It is only the ‘before the law’ consciousness that will prompt a deferential approach to the demands of legality. And just as in relation to families, we must explore the conditions under which particular narratives become salient for medical staff. What role do internal features of the medical organisation, such as legal advisors and complaints processes, play here? In relation to external features, Halliday has argued that the legal compliance of public bodies may be governed through a combination of hierarchical, community and market mechanisms.^70^ In what ways, then, do regulators, auditors and courts shape the legal consciousness of medical staff? What role do professional organisations play, for example, in promoting or resisting legal values as professional values?^71^ What capacity does the market have in this field to influence the perspectives of individual staff members and teams? All of these questions are important for a full understanding of law in this important domain of life and death – and everything in between.

## Footnotes

* We are grateful to Kathy Cerminara, Sharon Cowan, Sarah Nettleton, Julie Latchem and Glenys Williams, all of whom read and commented on earlier drafts. This paper was part-funded by Research Priming Funds provided by the Wellcome Trust [ref: 097829/Z/11/A].

1 B Jennett and F Plum ‘Persistent vegetative state after brain damage: a syndrome in search of a name’ (1972) 1 The Lancet (7753) 734.

2 JT Giacino et al ‘The minimally conscious state: definition and diagnostic criteria’ (2002) 58 Neurology 349.

3 DN MacCormick ‘Four quadrants of jurisprudence’ in W Kravitz, GH von Wright and DN MacCormick (eds) *Prescriptive Formality and Normative Rationality in Modern Legal Systems: Essays in Honour of R.S. Summers* (Berlin: Duncker and Humbolt, 1994);C McCrudden ‘Legal research and the social sciences’ (2006) 122 Law Q Rev 632;S Halliday (ed) *An Introduction to the Study of Law* (Edinburgh: W Greens, 2012).

4 See eg M Donnelly ‘Best interests, patient participation and the Mental Capacity Act 2005’ (2009) 17 Med L Rev 1;M Donnelly ‘Determining best interests under the Mental Capacity Act 2005’ (2011) 19 Med L Rev 304.

5 See eg A Grubbe ‘Persistent vegetative state: the duty (not) to treat and conscientious objection’ (1997) 4 Eur J Health L 157;P Lewis ‘Withdrawal of treatment from a patient in a permanent vegetative state: judicial involvement and innovative “treatment” ’ (2007) 15 Med L Rev 392;M Davies et al ‘Withdrawing and withholding artificial nutrition and hydration from a patient in a minimally conscious state (*Re M*)’ (2012) 38 J Med Ethics 64.

6 G Pradella ‘Substituting a judgment of best interests: dignity and the application of objective principles to PVS cases in the UK’ (2005) 12 Eur J Health L 335;S Rode ‘End-of-life decisionmaking for patients in persistent vegetative states: a comparative analysis’ (2007) 30 Hastings Int'l & Comp L Rev 477;R Jox et al ‘Substitute decision making in medicine: comparative analysis of the ethico-legal discourse in England and Germany’ (2008) 11 Medical Health Care & Phil 153.See also D Mendelson and T Stoltzfus ‘A comparative study of the law of palliative care and end-of-life treatment’ (2003) 31 J L Med & Ethics 130.

7 See eg J Finnis ‘*Bland*: crossing the Rubicon?’ (1993) 109 Law Q Rev 329;J Keown ‘Restoring moral and intellectual shape to the law after *Bland*’ (1997) 113 Law Q Rev 481;A McGee ‘Finding a way through the ethical and legal maze: withdrawal of medical treatment and euthanasia’ (2005) 13 Med L Rev 357;J Keown ‘A futile defence of *Bland*; a reply to Andrew McGee’ (2005) 13 Med L Rev 393;M Ford ‘The personhood paradox and the right to die’ (2005) 13 Med L Rev 80;MC Dunn et al ‘Constructing and reconstructing best interests: an interpretative examination of substitute decision-making under the Mental Capacity Act 2005’ (2007) 29 J Soc Welfare & Fam L 117;J Coggon ‘Ignoring the moral and intellectual shape of the law after *Bland:* the unintended side-effect of a sorry compromise’ (2007) 27 Legal Stud 110;L Skene et al ‘Neuroimaging and the withdrawal of life-sustaining treatment from patients in vegetative state’ (2009) 17 Med L Rev 245.

8 A Grubbe et al ‘Survey of British clinicians' views on management of patients in persistent vegetative state’ (1996) 248 The Lancet 35;K Dierickx et al ‘Belgian doctors' attitudes on the management of patients in persistent vegetative state: ethical and regulatory aspects’ (1998) 140 Acta Neurochir 481.

9 See eg M Hertogh ‘A “European” conception of legal consciousness: rediscovering Eugen Ehrlich’ (2004) 31 J Legal Stud 455;S Silbey ‘After legal consciousness’ (2005) 1 Ann Rev L & Soc Sci 323;M Kurkchiyan ‘Perceptions of law and social order: a cross-national comparison of collective legal consciousness’ (2011) 29 Wis Int'l L J 102.

10 SE Merry *Getting Justice and Getting Even: Legal Consciousness among Working-Class Americans* (Chicago: The University of Chicago Press, 1990);A Sarat ‘ “… the law is all over:” power, resistance, and the legal consciousness of the welfare poor’ (1990) 2 Yale J L & Humans 343;D Engel ‘How does law matter in the constitution of legal consciousness?’ in B Garth and A Sarat (eds) *How Does Law Matter?* (Evanston, IL: Northwestern University Press, 1998);N Mezey ‘Out of the ordinary: law, power, culture and the commonplace’ (2001) 26 Law & Soc Inquiry 145;D Cowan ‘Legal consciousness: some observations’ (2004) 67 Mod L Rev 928.

11 S Halliday and B Morgan ‘I fought the law and the law won? Legal consciousness and the critical imagination’ (2013) 66 Current Legal Probs 1.

12 P Ewick and S Silbey *The Common Place of Law: Stories from Everyday Life* (Chicago: The University of Chicago Press, 1998).

13 A Sarat and T Kearns (eds) *Law in Everyday Life* (Ann Arbor, MI: University of Michigan Press, 1995).

14 See generally MA Jones *Medical Negligence* (London: Sweet & Maxwell, 2008).

15 Lack of mental capacity is defined in ss 2 and 3.

16 Currently, only 4% of the population of England and Wales reports having made an Advance Decision and only 4% reports having appointed anyone as their Health and Welfare Lasting Power of Attorney (YouGov 2013; http://www.compassionindying.org.uk/knowledge-end-life-rights-and-choices-yougov-poll2013, accessed 15 January 2014). In *G* *v* *E* [2010] EWCA 2512, J Baker elaborated the principle underpinning the statutory provisions regarding deputies: ‘the words of s16(4) are clear. They do not permit the court to appoint deputies simply because “it feels confident it can” but only when satisfied that the circumstances and the decisions which will fall to be taken will be more appropriately taken by a deputy or deputies rather than by a court, bearing in mind the principle that decisions by the courts are to be preferred to decisions by deputies’ (para 61).

17 Mental Capacity Act 2005, s 24.

18 Ibid, ss 9–11. Note, however, that, under s 11(8) an attorney can only refuse life-sustaining treatment if the grant of the power of attorney expressly provides for this.

19 Ibid, s 16.

20 Ibid, s 20.

21 Albeit wrongly, 48% of people believe that they have the legal right to make medical decisions on behalf of an adult family member who lacks capacity to make decisions for themselves; 22% did not know whether they had this legal right or not; only 22% answered correctly that they did not have this right (YouGov 2013; http://www.compassionindying.org.uk/knowledge-end-life-rights-and-choices-yougov-poll2013, accessed 15 January 2014).

22 Mental Capacity Act 2005, s 4.

23 Court of Protection Practice Direction 9E, available at http://webarchive.nationalarchives.gov.uk/20110218200720/http://www.hmcourts-service.gov.uk/cms/files/09E_-_Serious_Medical_Treatment_PD.pdf (accessed 15 January 2014).

24 *Airedale NHS Trust* *v* *Bland* [1993] AC 789;*W* *v* *M and Others* [2011] EWHC 2443 (Fam).

25 *Aintree University Hospitals NHS Foundation Trust* *v* *James* [2013] UKSC 67, per Lady Hale, at para 22.

26 See above n 6.

27 Mental Capacity Act 2005, s 4.

28 The ‘best interests’ test is equally applicable, of course, to more routine clinical decisions not involving ‘serious medical treatment’.

29 For critical reflections on the *W* *v* *M* decision, seeA Mullock ‘Deciding the fate of a minimally conscious patient: an unsatisfactory balancing act?’ (2012) 20 Med L Rev 460; E Jackson ‘The minimally conscious state and treatment withdrawal: *W v M*’ (2013) 39 J Med Ethics 17.

30 *W* *v* *M and Others* [2011] EWHC 2443 (Fam).

31 Further anonymising (including reassigning pseudonyms – and occasionally altering identifying details, e.g. gender of speakers/patient or the cause of the injury) became necessary at the point at which presentations and publications were prepared. The challenges of avoiding ‘jigsaw identification’ of participants across our publications and of maintaining the confidentiality of those whose stories may also be in the public domain following court hearings and media interest is discussed in B Saunders, C Kitzinger and J Kitzinger ‘Anonymising interviews for data sharing: the practical research ethics of protecting participant identities’ European Sociological Association Conference, Turin, Italy, 2013.

32 For more information about interviewees (and patients) represented by the sample, see ibid.

33 P Bourdieu ‘The forms of capital’ in J Richardson (ed) *The Handbook of Theory and Research for the Sociology of Education* (New York: Greenwood Press, 1986).

34 A Sarat and T Kearns ‘Beyond the great divide: forms of legal scholarship and everyday life’ in Sarat and Kearns, above n 13;K Levine and V Mellema ‘Strategizing the street: how law matters in the lives of women in the street-level drug economy’ (2001) Law & Soc Inquiry 169.

35 See Ewick and Silbey, above n 12.

36 A Giddens *The Consequences of Modernity* (Cambridge: Polity Press, 1990).

37 For survey data about levels of trust in the medical profession, see MW Calnan and E Sanford ‘Public trust in health care: the system or the doctor?’ (2004) 13 Qual Saf Health Care 92;C Tarrant et al ‘Factors associated with patients' trust in their general practitioner: a cross-sectional survey’ (2003) 53 Br J Gen Prac 798. For a discussion of trust in the context of euthanasia, seeE Jackson ‘Death, euthanasia and the medical profession’ in B Brooks-Gordon et al *Death Rights and Rights* (Oxford: Hart Publishing, 1997).

38 P Sztompka *Trust: A Sociological Theory* (Cambridge, UK: Cambridge University Press, 1999) p 13.

39 The fact, for example, that death for a permanently vegetative patient may only be possible/allowed via the withdrawal of artificial nutrition and hydration is one potential source of tension between family members and care providers.

40 In this way, our interviewees' individual assertions of expertise match similar communal assertions arising from organised political/social movements. See eg S Epstein *Impure Science: AIDS, Activism and the Politics of Knowledge* (Berkeley, CA: University of California Press, 1996).

41 Sztompka, above n 38;see also N Luhmann ‘Familiarity, confidence, trust: problems and alternatives’ in D Gambetta (ed) *Trust: Making and Breaking of Co-operative Relations* (Oxford: Blackwell, 1988).

42 For further discussion, see Saunders et al, above n 31.

43 Mental Capacity Act 2005, s 24.

44 *Airedale NHS Trust* *v* *Bland* [1993] AC 789; Court of Protection Practice Direction 9E, available at http://webarchive.nationalarchives.gov.uk/20110218200720/http://www.hmcourts-service.gov.uk/cms/files/09E_-_Serious_Medical_Treatment_PD.pdf (accessed 15 January 2014).

45 See *W* *v* *M and Others* [2011] EWHC 2443 (Fam).

46 A classic UK example would be H Genn *Paths to Justice: What People Do and Think about Going to Law* (Oxford: Hart Publishing, 1999);H Genn and A Paterson *Paths to Justice in Scotland: What People Do and Think about Going to Law* (Oxford: Hart Publishing, 2000).

47 Interview with Ewick and Silbey in S Halliday and P Schmidt (eds) *Conducting Law & Society Research: Reflections on Methods and Practices* (New York: Cambridge University Press, 2009).

48 Ewick and Silbey, above n 12.

49 Halliday and Morgan, above n 11.

50 Ewick and Silbey, above n 12.

51 Halliday and Morgan, above n 11.

52 See eg M Douglas ‘Introduction to group-grid analysis’ in M Douglas (ed) *Essays in the Sociology of Perception* (London: Routledge and Kegan Paul, 1982);M Douglas ‘Cultural bias’ in M Douglas (ed) *In the Active Voice* (London: Routledge and Kegan Paul, 1982); M Thompson, R Ellis and A Wildavsky *Cultural Theory* (Boulder, CO: Westview Press, 1990).

53 Halliday and Morgan's argument is the relationship between these four ‘ideal types’ of legality are structured by the combination of ‘grid’ and ‘group’ outlined by Douglas.

54 Ewick and Silbey, above n 12, p 76.

55 Ibid, p 84.

56 [2011] EWHC 2443 (Fam).

57 Ewick and Silbey, above n 12, pp 186–187.

58 Halliday and Morgan, above n 11.

59 Sarat, above n 10;Cowan, above n 10.

60 E Hoffman ‘Legal consciousness and dispute resolution: different disputing behavior at two similar taxicab companies’ (2003) 26 Law & Soc Inquiry 691.

61 R Harding *Regulating Sexuality: Legal Consciousness in Lesbian and Gay Lives* (London: Routledge, 2011).

62 M Boittin ‘New perspectives from the oldest profession: abuse and the legal consciousness of sex workers in China’ (2013) 47 Law & Soc'y Rev 245.

63 Halliday and Morgan, above n 11.

64 K Bumiller *The Civil Rights Society: The Social Construction of Victims* (Baltimore, MD: Johns Hopkins University Press, 1988);Sarat and Kearns, above n 13;Ewick and Silbey, above n 12;LB Nielsen ‘Situating legal consciousness: experiences and attitudes of ordinary citizens about law and street harassment’ (2000) 34 Law & Soc Rev 1055;D Engel and F Munger *Rights of Inclusion: Law and Identity in the Life Stories of Americans with Disabilities* (Chicago, IL: The University of Chicago Press, 2003).

65 Silbey, above n 9.

66 For an overview, see E Rubin ‘Passing through the door: social movement literature and legal scholarship’ (2001) 150 U Pa L Rev 1.

67 See eg S Epstein *Impure Science: AIDS, Activism and the Politics of Knowledge* (Berkeley, CA: University of California Press, 1996).

68 Halliday and Morgan, above n 11.

69 A particularly stark example of the subversion of law by an ‘underground’ of medical professionals can be found in R Magnusson *Angels of Death: Exploring the Euthanasia Underground* (New Haven, CT: Yale University Press, 2002).

70 S Halliday ‘The governance of compliance with public law’ (2013) Pub L 312–324.

71 See C Epp *Making Rights Real: Activists, Bureaucrats and the Creation of the Legalistic State* (Chicago, IL: The University of Chicago Press, 2009).

